# Evaluation of Hospice Enrollment and Family and Unpaid Caregivers’ Experiences With Health Care Workers in the Care of Older Adults During the Last Month of Life

**DOI:** 10.1001/jamanetworkopen.2020.3599

**Published:** 2020-04-29

**Authors:** Jennifer L. Wolff, Vicki A. Freedman, Katherine A. Ornstein, John F. Mulcahy, Judith D. Kasper

**Affiliations:** 1 Department of Health Policy and Management, Roger C. Lipitz Center for Integrated Health Care, Johns Hopkins Bloomberg School of Public Health, Baltimore, Maryland; 2 Institute for Social Research, University of Michigan, Ann Arbor; 3 Icahn School of Medicine, Department of Geriatrics and Palliative Medicine, Mount Sinai, New York, New York

## Abstract

This survey study assesses end-of-life caregivers’ experiences with health care workers during older adults’ last month of life.

## Introduction

The care needs of older adults nearing end of life are extensive and variable, commonly encompassing help with self-care, medical tasks, symptom management, and high-stakes decisions.^1^ Although this demanding work is typically undertaken by family caregivers^1,2^ and increasingly occurs in partnership with hospice care,^3^ little is known about the experience of end-of-life and other caregivers when interacting with health care workers and whether end-of-life caregiving differs based on older adults’ use of hospice. This study draws on national survey data to assess end-of-life caregivers’ experiences in older adults’ last month of life.

## Methods

We examined the 2017 National Health and Aging Trends Study (NHATS), a survey of Medicare beneficiaries aged 65 years and older, and the National Study of Caregiving (NSOC), which interviews caregivers of NHATS participants.^4^ Participants in NHATS provided written consent, and participants in NSOC provided verbal consent. Both surveys followed American Association for Public Opinion Research (AAPOR) reporting guideline, which includes partial interviews. Both surveys were approved by the institutional review board at the Johns Hopkins Bloomberg School of Public Health. We assessed measures that have been previously identified as important to caregiver experiences when interacting with health care workers^5^ and during end-of-life care.^6^ Between-group differences were tested using 1-tailed Rao-Scott χ^2^ tests, with statistical significance set at *P* < .05. Analyses were completed using SAS version 9.4 (SAS Institute). We applied design variables and report weighted estimates that account for different probabilities of selection and response.

## Results

We examined responses from 1374 NSOC respondents providing self-care or mobility assistance to older adults living in residential care or community settings in 2017. Of an estimated 11.4 million family and unpaid caregivers, 2.5 million (21.5%) assisted during the last month of life. End-of-life and other caregivers reported that older adults’ health care workers always or usually listened (87.8% [SE, 2.5%] and 89.7% [SE, 1.6%], respectively; *P* = .41) and asked about their understanding of older adults’ health treatments (82.8% [SE, 3.2%] and 72.2% [SE, 3.0%], respectively; *P* = .02) (Figure). End-of-life caregivers were more likely than other caregivers to report being always or usually asked about needing help managing older adults’ treatments (47.0% [SE, 4.8%] and 29.9% [SE, 2.9%], respectively; *P* = .001); 1 in 3 end-of-life caregivers (32.3% [SE, 4.2%]) were never asked.

**Figure.**
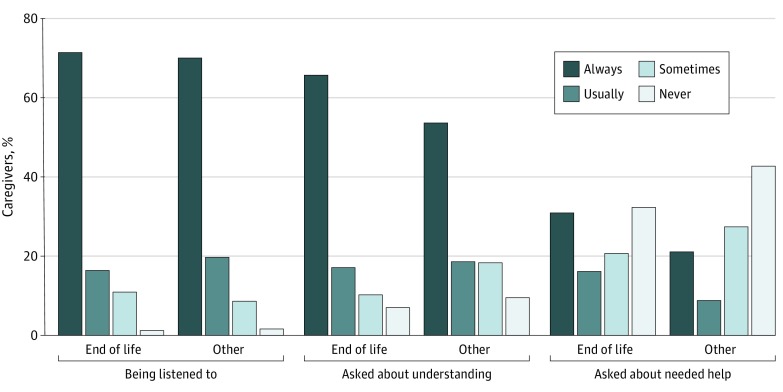
End-of-Life and Other Caregivers’ Experiences When Interacting With Older Adults’ Health Care Workers Estimates were weighted. Sample included family and other unpaid caregivers providing self-care or mobility help to an older adult in the last month of life (end-of-life caregivers) and those providing help to an older adult in the last month (other caregivers). Sample was limited to the two-thirds of end-of-life caregivers (1.6 million of 2.5 million [64.0%] weighted) and nearly half of other caregivers (4.3 million of 9.0 million [48.5%] weighted) who reported that they spoke with or emailed older adults’ health care workers in the past year. The unweighted proportion of end-of-life and other family and unpaid caregivers reporting their experiences of being listened to was 194 of 287 and 572 of 1084, respectively; of being asked about understanding, 194 and 571, respectively; and of being asked about needing help managing older adults’ treatments, 193 and 565, respectively.

Nearly half (48.0% [SE, 4.5%]) of end-of-life caregivers assisted an older adult who received 3 or more days of hospice (Table). Few differences in caregiver experiences were observed based on older adults’ hospice use. When reflecting on the last month of life, most caregivers reported being always or usually treated with respect (94.3% [SE, 1.7%]) and kept informed about older adults’ conditions (91.7% [SE, 1.8%]). Approximately 2 in 3 end-of-life caregivers (69.0% [SE, 3.4%]) reported talking with older adults about preferred medical care. More than half of end-of-life caregivers (51.4% [SE, 3.6%]) made medical decisions; approximately 1 in 10 reported decisions were made that they did not want (8.5% [SE, 1.7%]) or without enough input (9.5% [1.9%]). End-of-life caregivers commonly managed symptoms of anxiety or sadness (76.6% [SE, 3.9%]), pain (54.6% [SE, 4.3%]), and breathing (37.1% [SE, 3.8%]); these caregivers were more likely to receive training if the older adult was enrolled in hospice (56.2% [SE, 4.9%] vs 42.0% [SE, 3.9%]; *P* = .03).

**Table.** End-of-Life Caregivers' Experiences, Stratified by Older Adults' Hospice Use, Based on 2017 National Health and Aging Trends Study and 2017 National Study of Caregiving

Caregivers assisting in the last month of life^a^	All End-of-life caregivers (N = 287)		No hospice (n = 158)		Hospice (n = 129)^b^		*P* value
	No. (%)	SE	No. (%)	SE	No. (%)	SE	
Estimated No. in millions	2.5	NA	1.3	NA	1.2	NA	NA
Interactions with health care workers							
Always or usually treated with respect^c^	269 (94.3)	1.7	145 (91.9)	2.4	124 (96.8)	1.7	.07
Always or usually kept informed about older adults’ condition^c^	256 (91.7)	1.8	137 (91.3)	1.8	119 (92.2)	3.2	.81
Medical decision-making							
Talked with older adult about medical care wanted or not wanted	206 (69.0)	3.4	118 (73.2)	4.2	88 (64.4)	5.7	.22
Made medical decision in last month of life	163 (51.4)	3.6	85 (48.8)	4.1	78 (54.1)	6.0	.47
Decisions made about care or treatment without enough input from you	33 (9.5)	1.9	19 (10.5)	2.7	14 (8.5)	2.6	.59
Decisions made about care or treatment you did not want	33 (8.5)	1.7	20 (8.3)	2.0	13 (8.6)	2.9	.95
Symptom management							
Helped manage pain	150 (54.6)	4.3	75 (48.0)	6.2	75 (61.8)	5.4	.08
Helped manage breathing	111 (37.1)	3.8	56 (34.0)	4.6	55 (40.6)	6.1	.39
Helped manage feelings of anxiety or sadness	215 (76.7)	3.9	117 (74.2)	4.3	98 (79.4)	5.3	.37
Received training^d^	124 (49.1)	3.0	55 (42.0)	3.9	69 (56.2)	4.9	.03

Abbreviation: NA, not applicable.

^a^ Item nonresponse was less than 3.0% for all questions; estimates are weighted.

^b^ Hospice use was categorized based on administrative claims reflecting at least 3 days of hospice use in any setting in the last year of life.

^c^ Being treated with respect and being kept informed were limited to the 285 caregivers who reported the question applied to them in older adults’ last month of life.

^d^ Receipt of training was limited to the 239 caregivers who helped with symptom management in older adults' last month of life.

## Discussion

This national survey study found that end-of-life caregivers commonly assisted with symptom management and participated in medical decision-making. End-of-life caregivers generally reported favorable experiences of communication with health care workers, but there were gaps: 1 in 3 were never asked by health care workers if they needed help managing care, and just half of caregivers who assisted with symptom management received training. While training was more common among caregivers assisting older adults enrolled in hospice, findings indicate room for improvement.

End-of-life care increasingly involves hospice and occurs in the community; however, concerns about delayed hospice enrollment and burdensome end-of-life care persist.^1,3,6^ This study does not provide insight into specific factors affecting caregiver experiences or hospice enrollment. Nevertheless, the findings substantiate the feasibility and importance of monitoring the experiences of family members, who are often heavily involved in end-of-life care.^2^ Enhanced assessment of the end-of-life caregiving experience will be possible via annual NSOC data collection, beginning in 2021.
